# Book Notices

**Published:** 1868-05-15

**Authors:** 


### *BOOK NOTICES.*

Atlas of Venereal Diseases. By A. Cullerier, Surgeon to the Hôpital Du Midi, Member of the Surgical Society of Paris, Chevalier of the Legion D'Honneur, etc. Translated from the French, with Notes and Additions, by Freeman J. Bumstead, M.D., Professor of Venereal Diseases in the College of Physicians and Surgeons, New York, etc. With about one hundred and fifty beautifully Colored Figures, on twenty-six plates. Philadelphia: Henry C. Lea. 1868. To be complete in Five Parts, $3 each. For sale by W. B. Keen & Co., 148 Lake Street, Chicago.

The reception of Part First of this splendid work was acknowledged on p. 213 of the Journal. Part Second, just received, more than sustains the high anticipations which the first awakened.

The artistic execution of the work does high credit to the publisher. A friend who is familiar with the original assures us that the plates in the American edition are, in every particular, equal if not superior to the transatlantic copy.

The JOURNAL confesses never to have seen more vivid portraitures of pathological changes than here given. When completed, we shall have the parts bound in morocco and gilt edged.

Professor Bumstead has discharged his duties as editor with great fidelity. Himself a "dualist," and Cullerier a "unitist," he nevertheless comments with perfect candor and the fairness of assured strength. His own views are set forth in the introduction, with a sketch of the History, Virulence, Contagion, Evolution, Inheritance and Pathological Anatomy of Syphilis.

Part I. of the text discusses blennorrhagia in the male, complications and treatment. Part II. continues discussion of this branch of the subject, with especially noticeable chapters on blennorrhagic ophthalmia and arthritis. These two sections alone are worth more than the cost of the complete work. Then follows a sketch of blennorrhagia in the female, with its complications, under the captions Vulvitis, Vaginitis, Metritis, Ovaritis, and Urethritis—a concentrated and yet exhaustive *resumé* of the subject.

Chapter III. develops the subject of vegetations, and is illustrated by plates of most exquisite (pathological) beauty. This fasciculus is concluded by the commencing chapter of the section on soft chancre, with the best illustrative plates we have ever seen. We await the completion of the work with extreme interest, and in the meanwhile strongly recommend it to all of our readers at all interested in the subject.

A COMPLETE LIST OF THE MUSCLES OF THE HUMAN BODY. By WILLIAM LITTLE, M.D., Chicago. A Chart, size 24 by 38, giving the name, origin, insertion, and use of all the muscles in the human body. Price 50 cents; mounted on rollers, $1 50.

Also, the Cranial Nerves, twelve pairs, giving the name, origin, foramen of exit, distribution, and function. Price 20 cents. Address William Little, M.D., 220 Indiana street, Chicago.

A MANUAL OF THE DISSECTION OF THE HUMAN BODY. By Luther Holden, F.R.C.S., Assistant Surgeon of, and Lecturer on Anatomy at St. Bartholomew's Hospital, London. With notes and additions by Erskine Mason, M.D., Demonstrator of Anatomy at the College of Physicians and Surgeons, and Surgeon to the Charity Hospital, New York. Illustrated with numerous wood engravings. New York: Robert M. DeWitt, Publisher, No. 13 Frankfort street; pp. 588. For sale by W. B. Keen & Co., 148 Lake street.

A much needed and excellent manual. The illustrations are clear and elaborate, the descriptions concise and perspicuous, and the general artistic and typographic execution of the book, of a high order of merit. The American editor has performed his duty with fidelity and sound judgment.

As a companion in the dissecting room, or to refresh the memory of the practitioner, we cordially commend it to our readers.

THERAPEUTICS AND MATERIA MEDICA. A Systematic Treatise on the Action and Uses of Medicinal Agents, including their Description and History. By Alfred Stillè, M.D., Professor of the Theory and Practice of Medicine, and of Clinical Medicine, in the University of Pennsylvania, etc., etc. Third edition, revised and enlarged. In two volumes. Philadelphia: Henry C. Lea, 1868. For sale by W. B. Keen & Co., 148 Lake street.

This work, of course, does not require introduction to the intelligent readers of the JOURNAL. The second edition has been for many months out of print, and the author has, in the interim, been busily occupied in the selection of every thing of substantial value among recent advances in the science and art of Therapeutics. The subjects now treated of for the first time, are: *Chromic Acid; Permanganate of Potassa; The Sulphites of Soda*, etc.; *Carbolic Acid; Nitrous Oxide; Rhigolene:* and *Calabar Bean.*

The article on *Bromine* has been re-written, and that on *Electricity* materially enlarged and brought up to the times.

It is unnecessary to say that this treatise ranks any yet published on the subject.

SANITARY INSTITUTIONS DURING THE AUSTRO-PRUSSIAN-ITALIAN CONFLICT. Conferences of the International Societies of Relief for Wounded Soldiers. An Essay on Ambulance Wagons. Universal Exhibition Rewards and Letters. Catalogue of the Author's Sanitary Collection. By THOMAS W. EVANS, M.D., Officer of the Legion of Honor; Surgeon-Dentist to the Emperor Napoleon III., and to the Emperor of Russia; United States Commissioner to the Universal Exhibition, etc., etc. Third Edition. Paris: Printed by Simon Raçon & Co., No. 1 Rue Erfurth. 1868. [Printed for private distribution.]

A beautifully printed volume of 257 pages, replete with interesting and valuable information on the important subjects of which the title gives notice. Our sincere thanks are tendered the author for this addition to our library. A private note from Dr. Evans informs us that he has made arrangements with John Wiley & Son, of New York City, to distribute the balance of the edition. Those wishing to obtain copies can address accordingly.

CULLERIER'S ATLAS OF VENEREAL DISEASES. Since the notice of reception of the first two parts on a previous page went to press, Part III. has been received, but too late for comment in this No.

